# Supplementary material for: Modeling and Predicting Pulmonary Tuberculosis Incidence and Its Association with Air Pollution and Meteorological Factors Using an ARIMAX Model: An Ecological Study in Ningbo of China
Source: Int J Environ Res Public Health. 2022 Apr 28;19(9):5385. doi: 10.3390/ijerph19095385 (PMC9105987; doi:10.3390/ijerph19095385)
Supplement: Supplementary file 1 [file ijerph-19-05385-s001.zip › ijerph-1666768-supplementary.pdf]

Supplementary Materials

File S1: R code.

```
data="C:/cyp/fjh.csv"
library(forecast)
sjxldata=read.table(file = data,header = TRUE,sep = ",")

##Table1
summary(sjxldata)
library(psych)
describe(sjxldata)
library(pastecs)
stat.desc(sjxldata)

##Fig1
Sys.setlocale("LC_TIME","English")
library(reshape2)
library(ggplot2)
data2=read.csv("C:/cyp/fjh2.csv")
data2=data2[c(1:60),]
data2$date=as.Date(data2$date)
df=melt(data2,id="date")
names(df)=c("Time","variable","Value")
datebreak=seq(as.Date("2015-01-01"),as.Date("2019-12-01"),by="3 months")
ggplot(df,aes(x=Time,y=Value,color=variable))+
  geom_line(size=1.3)+
  facet_grid(variable~.,scales = "free")+
  theme(legend.position = "none")+
  scale_x_date(breaks = datebreak,date_labels="%b-%Y")+
  theme(axis.text.x = element_text(angle=45,hjust=1))

##Table 2 and Table 3
fbl1519=ts(sjxldata[,"fbl"],start=c(2015,1),end=c(2019,12),frequency = 12)
fbl1518=window(fbl1519,start=c(2015,1),end=c(2018,12))
Box.test(fbl1518,type="Ljung-Box")
fbl2019=window(fbl1519,start=c(2019,1),end=c(2019,12))

fitfbl1518=auto.arima(fbl1518,allowdrift = F,ic="bic",trace = T)
summary(fitfbl1518)
plot(fbl1518)
library(stats)
```

```
plot(stl(fbl1518,"per"))
Box.test(fitfbl1518$residuals,type="Ljung-Box")

PM2.51519=ts(sjxldata[,"PM2.5"],start=c(2015,1),end=c(2019,12),frequency = 12)
PM2.51518=window(PM2.51519,start=c(2015,1),end=c(2018,12))
fitPM2.51518=auto.arima(PM2.51518,allowdrift = F,ic="bic",trace = T)
Box.test(fitPM2.51518$residuals,type="Ljung-Box")
ccfpm2.5=ccf(fitfbl1518$residuals,fitPM2.51518$residuals)

PM101519=ts(sjxldata[,"PM10"],start=c(2015,1),end=c(2019,12),frequency = 12)
PM101518=window(PM101519,start=c(2015,1),end=c(2018,12))
fitPM101518=auto.arima(PM101518,allowdrift = F,ic="bic",trace = T)
Box.test(fitPM101518$residuals,type="Ljung-Box")
ccfPM10=ccf(fitfbl1518$residuals,fitPM101518$residuals)

SO21519=ts(sjxldata[,"SO2"],start=c(2015,1),end=c(2019,12),frequency = 12)
SO21518=window(SO21519,start=c(2015,1),end=c(2018,12))
fitSO21518=auto.arima(SO21518,,allowdrift = F,ic="bic",trace = T)
Box.test(fitSO21518$residuals,type="Ljung-Box")
ccfso2=ccf(fitfbl1518$residuals,fitSO21518$residuals)

CO1519=ts(sjxldata[,"CO"],start=c(2015,1),end=c(2019,12),frequency = 12)
CO1518=window(CO1519,start=c(2015,1),end=c(2018,12))
fitCO1518=auto.arima(CO1518,allowdrift = F,ic="bic",trace = T)
Box.test(fitCO1518$residuals,type="Ljung-Box")
ccfCO=ccf(fitfbl1518$residuals,fitCO1518$residuals)

NO21519=ts(sjxldata[,"NO2"],start=c(2015,1),end=c(2019,12),frequency = 12)
NO21518=window(NO21519,start=c(2015,1),end=c(2018,12))
fitNO21518=auto.arima(NO21518,allowdrift = F,ic="bic",trace = T)
Box.test(fitNO21518$residuals,type="Ljung-Box")
ccfNO2=ccf(fitfbl1518$residuals,fitNO21518$residuals)

O31519=ts(sjxldata[,"O3"],start=c(2015,1),end=c(2019,12),frequency = 12)
O31518=window(O31519,start=c(2015,1),end=c(2018,12))
fitO31518=auto.arima(O31518,allowdrift = F,ic="bic",trace = T)
Box.test(fitO31518$residuals,type="Ljung-Box")
ccfO3=ccf(fitfbl1518$residuals,fitO31518$residuals)

MAT1519=ts(sjxldata[,"temper"],start=c(2015,1),end=c(2019,12),frequency = 12)
MAT1518=window(MAT1519,start=c(2015,1),end=c(2018,12))
fitMAT1518=auto.arima(MAT1518,allowdrift = F,ic="bic",trace = T)
Box.test(fitMAT1518$residuals,type="Ljung-Box")
ccfMAT=ccf(fitfbl1518$residuals,fitMAT1518$residuals)
```

```
MALT1519=ts(sjxldata[,"Ltemperature"],start=c(2015,1),end=c(2019,12),frequency = 12)
MALT1518=window(MALT1519,start=c(2015,1),end=c(2018,12))
fitMALT1518=auto.arima(MALT1518,allowdrift = F,ic="bic",trace = T)
Box.test(fitMALT1518$residuals,type="Ljung-Box")
ccfMALT=ccf(fitfbl1518$residuals,fitMALT1518$residuals)

MAHT1519=ts(sjxldata[,"Htemperature"],start=c(2015,1),end=c(2019,12),frequency = 12)
MAHT1518=window(MAHT1519,start=c(2015,1),end=c(2018,12))
fitMAHT1518=auto.arima(MAHT1518,allowdrift = F,ic="bic",trace = T)
Box.test(fitMAHT1518$residuals,type="Ljung-Box")
ccfMAHT=ccf(fitfbl1518$residuals,fitMAHT1518$residuals)

MAH1519=ts(sjxldata[,"averhumidity"],start=c(2015,1),end=c(2019,12),frequency = 12)
MAH1518=window(MAH1519,start=c(2015,1),end=c(2018,12))
fitMAH1518=auto.arima(MAH1518,allowdrift = F,ic="bic",trace = T)
Box.test(fitMAH1518$residuals,type="Ljung-Box")
ccfMAH=ccf(fitfbl1518$residuals,fitMAH1518$residuals)

visibility1519=ts(sjxldata[,"visibility"],start=c(2015,1),end=c(2019,12),frequency = 12)
visibilitytrain=window(visibility1519,start=c(2015,1),end=c(2018,12))
fitvisibility=auto.arima(visibilitytrain,allowdrift = F,ic="bic",trace = T)
Box.test(fitvisibility$residuals,type="Ljung-Box")
ccfvisibility=ccf(fitfbl$residuals,fitvisibility$residuals)

MAP1519=ts(sjxldata[,"pressure"],start=c(2015,1),end=c(2019,12),frequency = 12)
MAP1518=window(MAP1519,start=c(2015,1),end=c(2018,12))
fitMAP1518=auto.arima(MAP1518,allowdrift = F,ic="bic",trace = T)
Box.test(fitMAP1518$residuals,type="Ljung-Box")
ccfMAP=ccf(fitfbl1518$residuals,fitMAP1518$residuals)

MAS1519=ts(sjxldata[,"windspeed"],start=c(2015,1),end=c(2019,12),frequency = 12)
MAS1518=window(MAS1519,start=c(2015,1),end=c(2018,12))
fitMAS1518=auto.arima(MAS1518,allowdrift = F,ic="bic",trace = T)
Box.test(fitMAS1518$residuals,type="Ljung-Box")
ccfMAS=ccf(fitfbl1518$residuals,fitMAS1518$residuals)

##Table4
ccfMATPM2.5=ccf(fitMAT1518$residuals,fitPM2.51518$residuals)
ccfMATPM10=ccf(fitMAT1518$residuals,fitPM101518$residuals)
ccfMATSO2=ccf(fitMAT1518$residuals,fitSO21518$residuals)
ccfMATCO=ccf(fitMAT1518$residuals,fitCO1518$residuals)
ccfMATNO2=ccf(fitMAT1518$residuals,fitNO21518$residuals)
ccfMATO3=ccf(fitMAT1518$residuals,fitO31518$residuals)
```

```
ccfMAHTPM2.5=ccf(fitMAHT1518$residuals,fitPM2.51518$residuals)
ccfMAHTPM10=ccf(fitMAHT1518$residuals,fitPM101518$residuals)
ccfMAHTSO2=ccf(fitMAHT1518$residuals,fitSO21518$residuals)
ccfMAHTCO=ccf(fitMAHT1518$residuals,fitCO1518$residuals)
ccfMAHTNO2=ccf(fitMAHT1518$residuals,fitNO21518$residuals)
ccfMAHTO3=ccf(fitMAHT1518$residuals,fitO31518$residuals)

ccfMALTPM2.5=ccf(fitMALT1518$residuals,fitPM2.51518$residuals)
ccfMALTPM10=ccf(fitMALT1518$residuals,fitPM101518$residuals)
ccfMALTSO2=ccf(fitMALT1518$residuals,fitSO21518$residuals)
ccfMALTCO=ccf(fitMALT1518$residuals,fitCO1518$residuals)
ccfMALTNO2=ccf(fitMALT1518$residuals,fitNO21518$residuals)
ccfMALTO3=ccf(fitMALT1518$residuals,fitO31518$residuals)

ccfMAHPM2.5=ccf(fitMAH1518$residuals,fitPM2.51518$residuals)
ccfMAHPM10=ccf(fitMAH1518$residuals,fitPM101518$residuals)
ccfMAHSO2=ccf(fitMAH1518$residuals,fitSO21518$residuals)
ccfMAHCO=ccf(fitMAH1518$residuals,fitCO1518$residuals)
ccfMAHNO2=ccf(fitMAH1518$residuals,fitNO21518$residuals)
ccfMAHO3=ccf(fitMAH1518$residuals,fitO31518$residuals)

ccfMAPPM2.5=ccf(fitMAP1518$residuals,fitPM2.51518$residuals)
ccfMAPPM10=ccf(fitMAP1518$residuals,fitPM101518$residuals)
ccfMAPSO2=ccf(fitMAP1518$residuals,fitSO21518$residuals)
ccfMAPCO=ccf(fitMAP1518$residuals,fitCO1518$residuals)
ccfMAPNO2=ccf(fitMAP1518$residuals,fitNO21518$residuals)
ccfMAPO3=ccf(fitMAP1518$residuals,fitO31518$residuals)

ccfMASPM2.5=ccf(fitMAS1518$residuals,fitPM2.51518$residuals)
ccfMASPM10=ccf(fitMAS1518$residuals,fitPM101518$residuals)
ccfMASSO2=ccf(fitMAS1518$residuals,fitSO21518$residuals)
ccfMASCO=ccf(fitMAS1518$residuals,fitCO1518$residuals)
ccfMASNO2=ccf(fitMAS1518$residuals,fitNO21518$residuals)
ccfMASO3=ccf(fitMAS1518$residuals,fitO31518$residuals)

##Table5
fbl1618=window(fbl1519,start=c(2016,1),end=c(2018,12))
fitfbl1618=auto.arima(fbl1618,allowdrift = F,ic="bic",trace = T)
summary(fitfbl1618)
tfitfbl1618=abs(fitfbl1618$coef)/sqrt(diag(fitfbl1618$var.coef))
pt(tfitfbl1618,35,lower.tail = F)
fore2019=forecast(fitfbl1618,12)
accuracy(fore2019,fbl2019)
```

```
#PM2.5
PM2.5lag3=stats::lag(PM2.51519,-3)
PM2.5lag31619=window(PM2.5lag3,start=c(2016,1),end=c(2019,12))
PM2.5lag31618=window(PM2.5lag31619,start=c(2016,1),end=c(2018,12))
PM2.5lag319=window(PM2.5lag31619,start=c(2019,1),end=c(2019,12))

arimaxPM2.5lag31618=Arima(fbl1618,order = c(0, 0, 0),seasonal = c(1, 1, 0),xreg=PM2.5lag31618)
tarimaxPM2.5lag31618=abs(arimaxPM2.5lag31618$coef)/sqrt(diag(arimaxPM2.5lag31618$var.coef))
pt(tarimaxPM2.5lag31618,34,lower.tail = F)#34=36-1-1
summary(arimaxPM2.5lag31618)

forePM2.5lag319=forecast(arimaxPM2.5lag31618,xreg=PM2.5lag319,12)
accuracy(forePM2.5lag319,fbl2019)

#PM10
PM10lag3=stats::lag(PM101519,-3)
PM10lag31619=window(PM10lag3,start=c(2016,1),end=c(2019,12))
PM10lag31618=window(PM10lag31619,start=c(2016,1),end=c(2018,12))
PM10lag319=window(PM10lag31619,start=c(2019,1),end=c(2019,12))

arimaxPM10lag31618=Arima(fbl1618,order = c(0, 0, 0),seasonal = c(1, 1, 0),xreg=PM10lag31618)
tarimaxPM10lag31618=abs(arimaxPM10lag31618$coef)/sqrt(diag(arimaxPM10lag31618$var.coef))
pt(tarimaxPM10lag31618,34,lower.tail = F)#34=36-1-1
summary(arimaxPM10lag31618)

forePM10lag319=forecast(arimaxPM10lag31618,xreg=PM10lag319,12)
accuracy(forePM10lag319,fbl2019)

#CO
COlag9=stats::lag(PM101519,-9)
COlag91619=window(COlag9,start=c(2016,1),end=c(2019,12))
COlag91618=window(COlag91619,start=c(2016,1),end=c(2018,12))
COlag919=window(COlag91619,start=c(2019,1),end=c(2019,12))

arimaxCOlag91618=auto.arima(fbl1618,d=0,D=1,max.p = 0,max.q =0,xreg=COlag91618,allowdrift = F,ic="bic",trace = T)
tarimaxCOlag91618=abs(arimaxCOlag91618$coef)/sqrt(diag(arimaxCOlag91618$var.coef))
pt(tarimaxCOlag91618,34,lower.tail = F)#34=36-1-1
```

```
summary(arimaxCOlag91618)

foreCOlag919=forecast(arimaxCOlag91618,xreg=COlag919,12)
accuracy(foreCOlag919,fbl2019)

#O3
O31618=window(O31519,start=c(2016,1),end=c(2018,12))
O319=window(O31519,start=c(2019,1),end=c(2019,12))

arimaxO31618=auto.arima(fbl1618,d=0,D=1,max.p = 0,max.q =0,xreg=O31618,allowdrift = F,ic="bic",trace = T)
tarimaxO31618=abs(arimaxO31618$coef)/sqrt(diag(arimaxO31618$var.coef))
pt(tarimaxO31618,34,lower.tail = F)#34=36-1-1
summary(arimaxO31618)

foreO319=forecast(arimaxO31618,xreg=O319,12)
accuracy(foreO319,fbl2019)

#MAT
MATlag1=stats::lag(MAT1519,-1)
MATlag11619=window(MATlag1,start=c(2016,1),end=c(2019,12))
MATlag11618=window(MATlag11619,start=c(2016,1),end=c(2018,12))
MATlag119=window(MATlag11619,start=c(2019,1),end=c(2019,12))

arimaxMATlag11618=auto.arima(fbl1618,d=0,D=1,max.p = 0,max.q =0,xreg=MATlag11618,allowdrift = F,ic="bic",trace = T)
tarimaxMATlag11618=abs(arimaxMATlag11618$coef)/sqrt(diag(arimaxMATlag11618$var.coef))
pt(tarimaxMATlag11618,34,lower.tail = F)#34=36-1-1
summary(arimaxMATlag11618)

foreMATlag119=forecast(arimaxMATlag11618,xreg=MATlag119,12)
accuracy(foreMATlag119,fbl2019)

MATlag3=stats::lag(MAT1519,-3)
MATlag31619=window(MATlag3,start=c(2016,1),end=c(2019,12))
MATlag31618=window(MATlag31619,start=c(2016,1),end=c(2018,12))
MATlag319=window(MATlag31619,start=c(2019,1),end=c(2019,12))

arimaxMATlag31618=auto.arima(fbl1618,d=0,D=1,max.p = 0,max.q =0,xreg=MATlag31618,allowdrift = F,ic="bic",trace = T)
tarimaxMATlag31618=abs(arimaxMATlag31618$coef)/sqrt(diag(arimaxMATlag31618$var.coef))
pt(tarimaxMATlag31618,34,lower.tail = F)#34=36-1-1
```

```
summary(arimaxMATlag31618)

foreMATlag319=forecast(arimaxMATlag31618,xreg=MATlag319,12)
accuracy(foreMATlag319,fbl2019)

#MALT
MALTlag1=stats::lag(MALT1519,-1)
MALTlag11619=window(MALTlag1,start=c(2016,1),end=c(2019,12))
MALTlag11618=window(MALTlag11619,start=c(2016,1),end=c(2018,12))
MALTlag119=window(MALTlag11619,start=c(2019,1),end=c(2019,12))

arimaxMALTlag11618=auto.arima(fbl1618,d=0,D=1,max.p = 0,max.q
=0,xreg=MALTlag11618,allowdrift = F,ic="bic",trace = T)
arimaxMALTlag11618=Arima(fbl1618,order = c(0, 0, 0),seasonal = c(1, 1,
0),xreg=MALTlag11618)
tarimaxMALTlag11618=abs(arimaxMALTlag11618$coef)/sqrt(diag(arimaxMALTlag11618$var
.coef))
pt(tarimaxMALTlag11618,34,lower.tail = F)#34=36-1-1
summary(arimaxMALTlag11618)

foreMALTlag119=forecast(arimaxMALTlag11618,xreg=MALTlag119,12)
accuracy(foreMALTlag119,fbl2019)

MALTlag3=stats::lag(MALT1519,-3)
MALTlag31619=window(MALTlag3,start=c(2016,1),end=c(2019,12))
MALTlag31618=window(MALTlag31619,start=c(2016,1),end=c(2018,12))
MALTlag319=window(MALTlag31619,start=c(2019,1),end=c(2019,12))

arimaxMALTlag31618=auto.arima(fbl1618,d=0,D=1,max.p = 0,max.q
=0,xreg=MALTlag31618,allowdrift = F,ic="bic",trace = T)
arimaxMALTlag31618=Arima(fbl1618,order = c(0, 0, 0),seasonal = c(1, 1,

0),xreg=MALTlag31618)#可不运行

tarimaxMALTlag31618=abs(arimaxMALTlag31618$coef)/sqrt(diag(arimaxMALTlag31618$var
.coef))
pt(tarimaxMALTlag31618,34,lower.tail = F)#34=36-1-1
summary(arimaxMALTlag31618)

foreMALTlag319=forecast(arimaxMALTlag31618,xreg=MALTlag319,12)
accuracy(foreMALTlag319,fbl2019)

#MAH
MAHlag7=stats::lag(MAH1519,-7)
MAHlag71619=window(MAHlag7,start=c(2016,1),end=c(2019,12))
```

```
MAHlag71618=window(MAHlag71619,start=c(2016,1),end=c(2018,12))
MAHlag719=window(MAHlag71619,start=c(2019,1),end=c(2019,12))

arimaxMAHlag71618=auto.arima(fbl1618,d=0,D=1,max.p = 0,max.q =0,xreg=MAHlag71618,allowdrift = F,ic="bic",trace = T)
arimaxMAHlag71618=Arima(fbl1618,order = c(0, 0, 0),seasonal = c(1, 1, 0),xreg=MAHlag71618)
tarimaxMAHlag71618=abs(arimaxMAHlag71618$coef)/sqrt(diag(arimaxMAHlag71618$var.coef))
pt(tarimaxMAHlag71618,34,lower.tail = F)#34=36-1-1
summary(arimaxMAHlag71618)

foreMAHlag719=forecast(arimaxMAHlag71618,xreg=MAHlag719,12)
accuracy(foreMAHlag719,fbl2019)

#MAP
MAPlag2=stats::lag(MAP1519,-2)
MAPlag21619=window(MAPlag2,start=c(2016,1),end=c(2019,12))
MAPlag21618=window(MAPlag21619,start=c(2016,1),end=c(2018,12))
MAPlag219=window(MAPlag21619,start=c(2019,1),end=c(2019,12))

arimaxMAPlag21618=auto.arima(fbl1618,d=0,D=1,max.p = 0,max.q =0,xreg=MAPlag21618,allowdrift = F,ic="bic",trace = T)
arimaxMAPlag21618=Arima(fbl1618,order = c(0, 0, 0),seasonal = c(1, 1, 0),xreg=MAPlag21618)
tarimaxMAPlag21618=abs(arimaxMAPlag21618$coef)/sqrt(diag(arimaxMAPlag21618$var.coef))
pt(tarimaxMAPlag21618,34,lower.tail = F)#34=36-1-1
summary(arimaxMAPlag21618)

foreMAPlag219=forecast(arimaxMAPlag21618,xreg=MAPlag219,12)
accuracy(foreMAPlag219,fbl2019)

MAPlag11=stats::lag(MAP1519,-11)
MAPlag111619=window(MAPlag11,start=c(2016,1),end=c(2019,12))
MAPlag111618=window(MAPlag111619,start=c(2016,1),end=c(2018,12))
MAPlag1119=window(MAPlag111619,start=c(2019,1),end=c(2019,12))

arimaxMAPlag111618=auto.arima(fbl1618,d=0,D=1,max.p = 0,max.q =0,xreg=MAPlag111618,allowdrift = F,ic="bic",trace = T)
arimaxMAPlag111618=Arima(fbl1618,order = c(0, 0, 0),seasonal = c(1, 1, 0),xreg=MAPlag111618)
tarimaxMAPlag111618=abs(arimaxMAPlag111618$coef)/sqrt(diag(arimaxMAPlag111618$var.coef))
pt(tarimaxMAPlag111618,34,lower.tail = F)#34=36-1-1
```

```
summary(arimaxMAPlag111618)

foreMAPlag1119=forecast(arimaxMAPlag111618,xreg=MAPlag1119,12)
accuracy(foreMAPlag1119,fbl2019)

#O3MAPlag11
xregO3MAPlag111618=cbind(O31618,MAPlag111618)
arimaxO3MAPlag111618=auto.arima(fbl1618,d=0,D=1,max.p = 0,max.q =0,xreg=xregO3MAPlag111618,allowdrift = F,ic="bic",trace = T)
tarimaxO3MAPlag111618=abs(arimaxO3MAPlag111618$coef)/sqrt(diag(arimaxO3MAPlag111618$var.coef))
pt(tarimaxO3MAPlag111618,33,lower.tail = F)#33=36-1-2
summary(arimaxO3MAPlag111618)

O31618=O319
MAPlag111618=MAPlag1119

xregO3MAPlag1119=cbind(O31618,MAPlag111618)
foreO3MAPlag1119=forecast(arimaxO3MAPlag111618,xreg=xregO3MAPlag1119,12)
accuracy(foreO3MAPlag1119,fbl2019)
```
